# From Nature to Design: Tailoring Pure Mycelial Materials for the Needs of Tomorrow

**DOI:** 10.3390/jof10030183

**Published:** 2024-02-28

**Authors:** Viraj Whabi, Bosco Yu, Jianping Xu

**Affiliations:** 1 Department of Biology, McMaster University, Hamilton, ON L8S 4K1, Canada; whabiv@mcmaster.ca; 2 Department of Mechanical Engineering, Engineering Office Wing, University of Victoria, Victoria, BC V8P 3E6, Canada; boscoyu@uvic.ca; 3 Department of Materials Science and Engineering, McMaster University, Hamilton, ON L8S 4L7, Canada

**Keywords:** biodegradable materials, foams, porous materials, mycelium-based leathers, mycelial films, strain optimization, hyphal structures, liquid-state surface fermentation, submerged fermentation, solid-state fermentation

## Abstract

Modern efforts to influence materials science with principles of biology have allowed fungal mycelial materials to take a foothold and develop novel solutions for the circular bioeconomy of tomorrow. However, recent studies have shown that the value of tomorrow’s green materials is not determined simply by their environmental viability, but rather by their ability to make the polluting materials of today obsolete. With an inherently strong structure of chitin and β-glucan, the ever-adaptable mycelia of fungi can compete at the highest levels with a litany of materials from leather to polyurethane foam to paper to wood. There are significant efforts to optimize pure mycelial materials (PMMs) through the entire process of species and strain selection, mycelial growth, and fabrication. Indeed, the promising investigations of novel species demonstrate how the diversity of fungi can be leveraged to create uniquely specialized materials. This review aims to highlight PMMs’ current trajectory, evaluate the successes in technology, and explore how these new materials can help shape a better tomorrow.

## 1. Towards Functional Fungi

The kingdom of fungi with its diverse portfolios of life cycles and adaptive morphology has been selectively cultivated since 600 AD [1]. Often characterized by unique cellular organizations, filamentous fungi consist of branching filaments called hyphae that form complex structures [2]. Composed of chitinous cell wall, the hyphae grow rapidly in networks, intaking nutrients to form a densely packed biomass known as mycelium [2,3,4]. Fungal mycelium has found recent attention from materials scientists due to its inherently robust structure which has been leveraged to create materials for construction, insulation, fashion, and other uses [5,6,7]. Combined with fungi’s ability to upcycle wastes into constructive mycelia, the fabrication of these functional mycelial materials is cheap, non-energy intensive, and, most importantly, renewable [8]. As such, there is growing interest, both academic and industrial, in this field of ‘fungineering’ and developing better materials on-route to the transition toward a circular bioeconomy [9].

Preliminary research into this niche fungal biotechnology began with composites of mycelia and agricultural by-products. As the fungal biomass grows through the substrate, the natural secretions of the mycelia interlink them together into a reinforced material that is stronger than either of its parts [10]. These composite mycelial materials (CMMs) have already been deployed industrially and are widely regarded as feasible competitors to more established polystyrene packaging materials at lower embodied energy and carbon emission levels [8]. The current and future prospects of CMMs have been discussed in detail in notable review papers in recent years (See reviews [11,12]).

On the other hand, a newer family of materials made purely from mycelia has emerged and shown promising room for growth. Rather than combining with the substrate, these pure mycelial materials (PMMs) are separated from the substrate after cultivation or, in a few cases, exhaust the substrate entirely [13]. Start-ups such as Mycotech, Ecovative LLC, and Quorn have made strides using pure mycelia towards replacing leathers, high-performance foams, and meat [14,15,16]. However, for broad adoption, there are significant challenges in making PMMs the premier choice over conventional products with greater market history. This review highlights recent advances in the field, discusses major knowledge gaps in optimizing PMMs, and evaluates current methods in overcoming these challenges.

## 2. Techniques in the Cultivation of Mycelium

**Figure 1:**
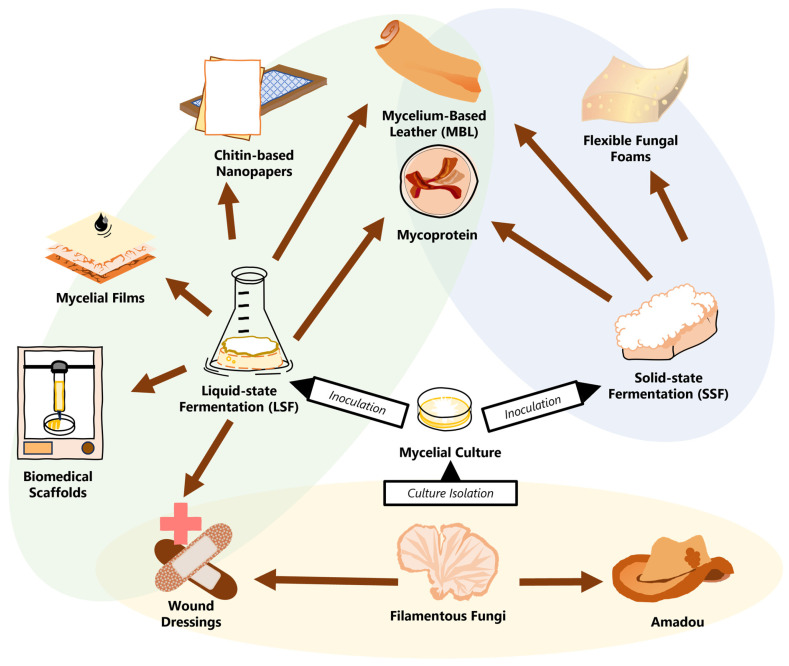
The current product portfolio of PMMs. From mushroom to mycelium, the cultures are isolated and then inoculated onto substrates. Functional materials (marked by brown arrows) are categorized by the type of fabrication, either directly from the fungal fruiting bodies (yellow) or with cultivated mycelial mats from LSF (green) or SSF (blue). Materials in overlapping sections can be made using multiple different techniques.

From industry to research, the growing interest in mycelial materials has necessitated evolution in fungiculture beyond targeting mushroom fruiting and toward maximizing hyphal production [17,18,19,20]. The various growing and processing methods offer specific advantages to create unique groups of materials with a wide scope of applications (as seen in Figure 1).

### 2.1. Solid-State Fermentation

Since ancient times, solid-state fermentation (SSF) has long been the ubiquitous method, as can be seen in the techniques making kōji from rice inoculated with *Aspergillus oryzae* or farming edible varieties of mushrooms [21,22,23,24,25]. The process involves inoculating grain or agricultural by-products with liquid fungal culture to create spawn that is left to fruit in fixed environmental conditions absent of free water [23,26]. Membrane filters made from cheesecloth or polycarbonate are sometimes used as a way to efficiently segregate the hyphal biomass from the substrate below; however, this can lead to reduced respiration rates [19,25]. Furthermore, careful consideration must be given to the diffusion of oxygen, as the formation of the intermediate wet hyphal layer can prevent oxygen from reaching the spawn and substrate below [25]. In mushroom farms, the solid substrate can live anew after harvest in the form of composites that use the combined strengths of the fungal hyphal network and the compacted substrate to create sustainable packaging and construction materials [5,12,22]. At PMMs, the spent substrate is not used, and it is instead separated from the mycelial mat that forms at the air/substrate interface [7,16,19]. Due to the high CO_2_ in the growing environment, the mycelia forms a fluffy layer of aerial hyphae that branches upward out of the substrate for oxygen [16,23]. The resultant mat can thicken and strengthen due to the protein-rich environment provided by the complex and diverse agricultural substrate [7,16,19].

Static tray-based SSF has recently risen as the more popular cultivation method for large-scale production of mycelia, with the two largest manufacturers, MycoWorks and Ecovative LLC, filing patents involving solid substrates [16,27,28,29]. Looking to optimize their tray-based SSF processes, companies can take inspiration from the more established mushroom farming industry for decisions on substrate selection, environmental conditions, and mass production [21,30]. MycoWorks, in particular, has been able to scale up their mycelium-based leather (MBL) production using self-contained, shallow trays filled with inoculated sawdust that are vertically stacked in order to maximize space during growth [29]. Similarly, Ecovative’s subsidiary My Foods has partnered with Canada-based Whitecrest Mushrooms Ltd. to develop “the world’s largest vertical mycelium farm” and increase the yield of their MyBacon mycoprotein [31].

### 2.2. Liquid-State Fermentation

Liquid-state fermentation (LSF) offers alternative processes wherein concentrated nutritional liquid (or mostly liquid) media with guaranteed nutrient profiles are homogeneous and different from the heterogenous solid substrates [17,18,19,20]. Common liquid media ingredients for wet lab applications such as potato dextrose broth, yeast malt dextrose broth, and blackstrap molasses are often used for the LSF of a variety of fungi [19,32,33,34]. Additionally, more complex solid substrate ingredients such as grains, straw, and sawdust can be homogenized with medium broth to create semi-solid slurries [7,18]. However, LSF processes are not monolithic, as the choice of fermentation conditions can lead to drastically different end-products.

One distinct process is called submerged fermentation (SmF) and involves bioreactors with vast volumes of axenic culture under constant agitation to create large quantities of mycelia [34,35]. For fungi, SmF is preferred as a more efficient way to derive bioactive metabolites from species such as *Cordyceps militaris*, *Inonotus obliquus,* and *Schizopyllum commune* as compared to the conventional SSF [34,36]. While the amount of mycelia produced in such a short time is remarkable, the biomass is often prone to microbial contamination [21]. Additionally, the constant agitation limits the morphology of mycelia to small pellets suitable for mycoproteins but nothing larger [15,35,37]. Yet, Finland’s VTT Technical Research Centre has shown in a pilot study that these disadvantages can be overcome with their novel MBL production. Their patent-pending SmF method utilizes a bioreactor to cultivate large quantities of mycelial slurry which is then dispensed at a rate of 1 m per minute before being dried, embossed, and processed [38].

A similar liquid-based method called liquid-state surface fermentation (LSSF) uses inoculated broth under static conditions to form mycelium at the interface between liquid and air. LSSF requires less overall energy than SmF and has the additional advantage of creating thick and tunable mycelial mats [13,20]. Fungal biomass derived from LSSF can be utilized in the creation of higher-order products such as MBLs as well as mycelial films, a new promising subgroup of PMMs that can be easily tuned to fit a wide range of functions such as binding agents, coatings, and membranes (See Section 3) [19,32,39]. Of note, LSSF has not yet been used for mass-scale production, but perhaps it could follow the vertical approach used by MycoWorks with assumedly cheaper costs attributed to lower material cost and less preparation. All current literature studies offer a limited perspective on LSSF, and it remains to be seen whether it can truly compare with either SmF or SSF at a wider scale. With their many differences, all of the methods are highly dependent on environmental conditions that need to be optimized for high yield and low variability. Optimizing these biotechnologies would offer the opportunity to tailor the performance of existing mycelial materials and pave the way towards many new ones [13,20]. These and potential future insights on cultivation substrates and conditions will result in PMMs with broad physical and chemical properties for increasingly broad applications.

## 3. The Growing Profile of Pure Mycelial Materials

### 3.1. Laying out the Design Space for PMMs

The recent interest in PMMs has steadily evolved into a large yet thoroughly uncharted collection of diverse materials the true potential of which is difficult to realize without an assay of the current prospects of these materials in their applications as leathers, foams, films, and more. One such method is through the process of materials selection, as introduced by Ashby and Cebon [40,41,42]. This process leverages materials data to systematically identify key qualities of comparable engineering materials in order to determine a desired materials profile that meets the necessary design function, objectives, and constraints [40,41,42]. The method of materials selection employs a measured approach in evaluating materials as they pertain to function, form, and design. To prioritize each one of these factors, the performance of materials is evaluated through relevant properties such as density or strength, as seen in Table 1, or a combination of many relevant properties in the form of a Material Property Index (MPI) [40,41,42]. The material to best fit the target application is the one which maximizes the optimization criteria of the MPI, while all others are ranked below in decreasing order [40,42].

The first step in crafting an MPI is to define an objective, typically minimum mass or density (ρ), which decides the direction of a design process. The next step comes with specific materials constraints as defined by the different components within the engineering design. For instance, materials like leathers need to be able to endure tensile conditions without reaching tensile rupture with a high enough ultimate tensile strength (σ_UTS_). MPI_1_ combines the minimum mass objective as well as the constraints on tensile performance to create an index to rank how MBLs perform in comparison to other leathers [42,43].

(1) $$\begin{array}{c} {\mathrm{MPI}}_{1}=\sigma_{\mathrm{UTS}}/\rho \end{array}\begin{array}{c} \mathrm{Objective}:\;\mathrm{minimize}\;\mathrm{mass}\;(\rho )\\ \mathrm{Constraint}:\;\mathrm{strong}\;\mathrm{enough}\;\mathrm{to}\;\mathrm{resist}\;\mathrm{tensile}\;\mathrm{rupture}\;(\sigma_{\mathrm{UTS}}) \end{array}$$

There are limits to this index, as leathers, along with rubbers, wools, and silks, do not only endure uniaxial tensile loads, but need to have an intrinsic springiness controlled by their stiffness or Young’s modulus (E) [43]. In line with the minimum mass design objective, MPI_2_ assesses the ability to store high amounts of energy before springing back without failure [40,43].

(2) $$\begin{array}{c} {\mathrm{MPI}}_{2}={\sigma_{\mathrm{UTS}}}^{2}/E\rho \end{array}\begin{array}{c} \mathrm{Objective}:\;\mathrm{minimize}\;\mathrm{mass}\;(\rho )\\ \mathrm{Constraint}:\;\mathrm{high}\;\mathrm{enough}\;\mathrm{elastic}\;\mathrm{energy}\;\mathrm{storage}\;({\sigma_{\mathrm{UTS}}}^{2}/E) \end{array}$$

**Figure 2:**
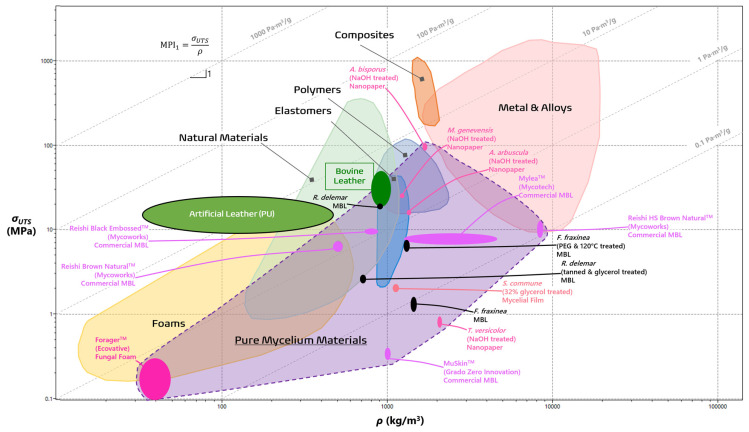
Material property chart comparing pure mycelial materials to typical materials families in terms of tensile strength (in MPa) against density (in kg/m^3^). The guideline signifies which materials optimize specific tensile strength with minimum mass designs. The image was generated using ANSYS, Inc. (https://www.ansys.com/, accessed on 20 January 2024).

**Figure 3:**
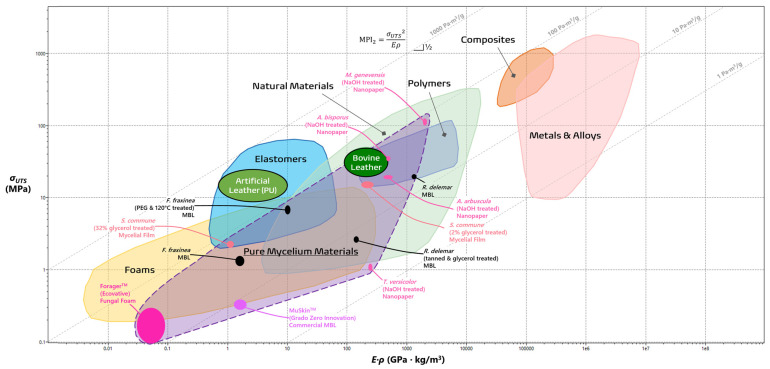
Material property chart comparing pure mycelial materials to typical materials families in terms of tensile strength (in MPa) against the product of density and Young’s modulus (in GPa·kg/m^3^). The guideline signifies which materials optimize energy absorption per unit mass. The image was generated using ANSYS, Inc. (https://www.ansys.com/, accessed on 20 January 2024).

In order to visually grade the performance of different materials, various combination of properties (e.g., σ_UTS_, E, ρ) for different materials are plotted using a material property chart (commonly known as an Ashby chart) in a log-log scale [40]. In Figure 2 and Figure 3, the wide gamut of PMMs can be visually compared to the typical material families [7,19,20,32,33,40,42,43,44,45,46,47,48,49,50,51]. Both MPI_1_ and MPI_2_ can be visualized as straight guidelines with defined slopes of 1 and ½, respectively. In the case for MPI_2_, for instance, materials higher on the line maximize the energy storage, while those that lie on it are on equal footing [40]. It is evident from the laid out purple space that PMMs fit comfortably within a wide range of material families, including foams, elastomers, and polymers, depending on their species of origin, treatment process, and intended functions. Leveraging the principles of materials selection by way of materials property charts and MPIs offers detailed performance metrics of novel materials like PMMs and outline the necessary trajectory for large-scale viability. This paper will evaluate each group of PMMs as they compare with the existing profile of material families in order to highlight their current strengths and illuminate how further development may help overcome their drawbacks.

### 3.2. The Past, Present, and Future of Mycelial Textiles

In contrast with the textile’s historical ubiquity, the current methods for modern leather production are becoming increasingly incompatible with society’s vision of a better future [19,44,52]. There is a coming paradigm shift towards vegan leather, with the industry projected to overtake the market for traditional leathers by 2027 [44,53]. The emergence of the more affordable MBLs is spearheading this paradigm shift towards sustainable alternatives, challenging the dominance of their bovine and synthetic counterparts [44,53]. A comprehensive life cycle assessment conducted on MycoWorks’ Reishi^TM^ MBL revealed promising environmental credentials [29]. In their 2022 pilot-scale production, mycelium-based leather boasted a remarkably low carbon footprint of 6.2 kg of CO_2_ equivalents per m^2^, a stark contrast to the 32.97 kg of CO_2_ equivalents per m^2^ associated with bovine leather [29]. As production scales up, projections suggest an increase to 13.88 kg of CO_2_ equivalents per m^2^; however, with optimized practices, this figure could plummet to as low as 2.76 kg of CO_2_ equivalents per m^2^ resulting from the transition to bio-gas free workflows [29]. Furthermore, research by Jones et al. highlights the superior cost-effectiveness of MBLs, with production costs estimated at a mere $0.18–0.28 per m^2^ compared to the substantially higher $5.38–6.24 per m^2^ for raw hides [44].

**Figure 4:**
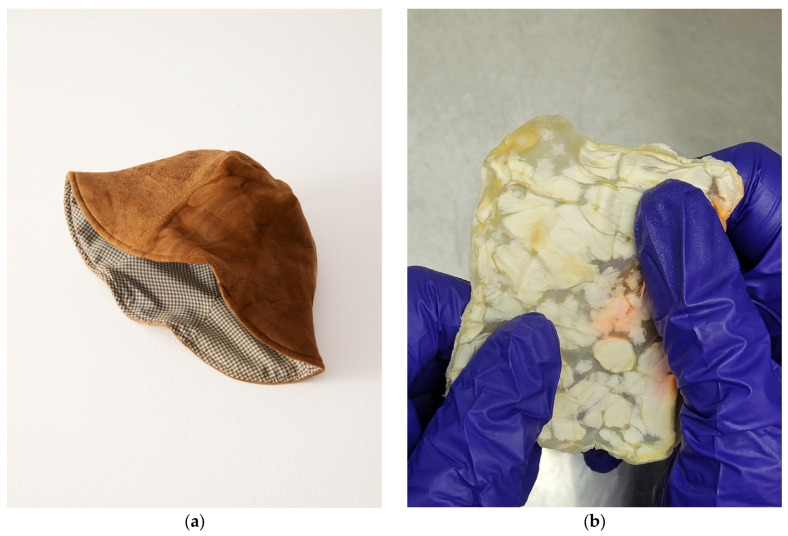
The product diversity of fungal textiles today. (**a**) The modern “Amadou tulip hat” made from the trama of *Fomes fomentarius* mushrooms sold by Eden Power Corp in 2022. (**b**) Lab-grown mycelium-based leather made from *Schizophyllum commune* mycelia grown through liquid-state fermentation and treated with polyethylene glycol.

Fabricating fungal textiles has a storied history with Transylvanian craftspeople utilizing mushrooms of *Fomes fomentarius* and *Piptoporus betulinus* to create Amadou leathers as early as the 19th century [54,55]. In their fabrication, wild fruiting bodies are collected by hand, and then boiled in caustic lye solutions to makes the process of fabrication smoother. From there, the material is trimmed to shape by following the natural “grain” or growth direction, and then stretched to create products such as hats, belts, bags, etc. [54,56]. The resulting finish is a breathable material similar to felt and close to the color of bovine leather as a result of the high composition of melanin-like substances [57]. On the other side of the world, a Tlingit wall pocket from 1903 was discovered to have made from similar mycelial textiles by indigenous communities in British Columbia [58]. Upon examining the hyphal morphology of the wall pocket with a scanning electron microscope (SEM), the mycelia were determined to be characteristic of *Fomitopsis officianalis*, another bracket fungi not too dissimilar to those employed in Transylvania. While the methods of the Tlingit community are unclear, the process of evaluating the global ethnomycological usage of fungi elucidates how best to recontextualize these textiles for today [54,58]. Amadou has not been left in history, however, with fashion houses (as seen in Figure 4a) and bush crafters alike finding ways to recontextualize the material to modern needs [59,60].

To meet modern levels of demand, the process of producing MBLs has become more efficient than the mushroom-based artisanal handcrafting of Amadou through a more industrial, fermentation-based cultivation of mycelia. SSF and LSSF offer better control on the quality of the mycelial mats compared to historical manufacturing that depended on the seasonality of foraged mushrooms [20,53,54]. After the cultivation period is over, the mycelial material is typically separated from the substrate and then subjected to a range of treatment procedures [44]. Before any cross-linking or physical treatment, the mycelial mats are pre-treated with hydrating agents such as glycerol, ethylene glycol, or polyethylene glycol (PEG) which also plasticize the hyphal fibers (as seen in Figure 4b) [19]. Next, the plasticized mats are immersed in alcohols or acetic acids in order to denature proteins and create sites for cross-linking [27,44]. Cross-linking with vegetable tannic acid allows the mycelia to more closely imitate the aesthetic, form, and function of conventional leathers [7,19,45,61]. In all cases, after chemical treatment, mechanical pressing of the materials is undertaken in an effort to further densify the mycelia with different methods using either heating or cooling to rapidly dry the mycelial mat [19,27,28].

According to investigations of the morphological, mechanical, and physiochemical characteristics of these materials, MBLs are more variable in comparison to bovine and synthetic leathers, as seen in Table 2 [18,19,62]. Comparing individual properties such as density, elongation rate, tensile strength, and Young’s modulus shows the different advantages of each material. While the tensile strengths of many MBLs are comparable to conventional leathers, the lower stiffness show that there is still a need to develop better post-processing methods for long-term feasibility [19,62]. Even those with high stiffnesses such as the treated *Rhizopus delemar* leather present failings with their poor elastic elongation rate [7].

Figure 2 visualizes materials that maximize their specific strength as defined with MPI_1_ as a guideline with a slope of 1. Bovine leather only boasts an MPI_1_ of 3.43 Pa·m^3^/g, while the artificial leather outperforms it by a whole order of magnitude [51,52,64,65]. The *F. fraxinea* leather improved impressively once cross-linked with PEG and heat pressed at 120 °C and even surpassed the reference bovine leather with an MPI_1_ of 4.9 Pa·m^3^/g [19]. On the other hand, the virgin *R. delemar*, the second best MBL, was more successful than its treated counterpart (2.2 Pa·m^3^/g vs. 0.378 Pa·m^3^/g respectively), demonstrating that not all species reap the same benefits from chemical treatments [62]. It is also worth noting that only two commercial MBLs, the Reishi^TM^ Brown Natural and Black Embossed, were in the same range as the bovine leathers, while all the rest lagged in this metric of specific strength.

Figure 3 visualizes materials with optimal energy storage per unit mass and optimal performances are defined in the region with MPI_2_ as a guideline and with a slope of ½. Successful leathers can store great amounts of energy and combine the properties of tensile strength and Young’s modulus in a ratio of σ_UTS_^2^/E [43]. Unfortunately, some commercial MBLs such as Reishi^TM^ and Mylea^TM^ do not have Young’s modulus data, excluding them from this analysis [46,47,48,49,50]. Artificial leathers derived from polyurethane are in their own league, as the majority of MBLs do not come close [19,64,65]. The lone outlier, Raman et al.’s treated *F. fraxinea* mycelia, builds on its excellent specific strength properties with an extraordinary elastic storage ability (MBI_2_ = 4990 Pa·m^3^/g) that even supersedes bovine leather [19,43]. The success of these treated MBLs highlights the importance of researching chemical cross-linking, heat treatment, and species-based optimization if these materials are to supplant the conventional leathers of today.

Other mechanical properties, such as large scratch recoveries and high dynamic stress resistance, demonstrate the capability of the textile to withstand continual, repetitive loads. Furthermore, the lack of external fungal and bacterial growth on fabricated MBLs demonstrates their natural antifungal and antibacterial properties [62]. Just recently, MBLs have evolved from a niche idea to a growing trend in sustainable fashion embodied by the products of brands such as Adidas, Balenciaga, and Hermès [13,66]. With significant knowledge gaps in optimizing mycelium mat cultivation and post-processing procedures, the success of MBLs is heavily reliant upon future research prospects and could further expand the applications of MBLs to fit the materials needs of tomorrow.

### 3.3. Flexible Fungal Foams

Flexible fungal foams are promising candidates to replace insulation, petroleum-based foams, and wood composite cores. Presently, Ecovative LLC’s patented Forager^TM^ is the sole pure mycelial biofoam on the market which is reported to be completely “tunable” in terms of tensile strength, density, and fiber orientation [67]. These materials are fabricated through SSF with the addition of a vented void chamber on top of a tray. Since the void chamber is only accessible through the vents, a CO_2_ gradient (3–7% concentration by volume) is introduced which encourages the mycelia to propagate through the vents and create an isolated mat of mycelia. Additionally, the relative humidity and temperature (29–35 °C) of the chamber are carefully chosen in order to mitigate primordial initiation which would compromise the mechanical properties of the foam. Before the foam is extracted, the mycelial mat is compressed to a chosen size and is left for an additional 72 h to densify and strengthen its fibers. Finally, the foam is separated from the substrate, dried at 43 °C, and, optionally, heat pressed to further densify the structure [16,68].

Presently, these foams are deployed as specialized textiles for the fashion industry that are marketed to be “insulating, water-repellent, and fire-resistant” [6,67,69]. Interestingly, a densified, closed-cell variety of Ecovative’s foams has been shown to work as an excellent acoustic shield at a wide frequency range from 350 Hz to 4 kHz [70]. With the widespread employment of mineral wools, synthetic fibers, and petrochemical-derived polyurethane foams, these flexible fungal foams shine as greener and more sustainable alternatives [6,71]. Since there is only one player on the market, plans to apply these uniquely adaptable foams are nascent. The purported tensile strength (0.1 to 0.3 MPa), Young’s modulus (0.6 to 2.0 MPa), and density (0.03 to 0.05 g/cm^3^) of the Forager^TM^ material shows that it has a place, albeit small, in the foam material family, as seen in Figure 2 and Figure 3 [20,68]. It performs worse than bovine and artificial leathers in terms of specific strength (MPI_1_ = 0.447 Pa·m^3^/g) and elastic energy storage (MPI_2_ = 707 Pa·m^3^/g). However, these numbers should be taken with caution as they do not come from any peer-reviewed measurements in original research papers and are instead reported in Gandia et al.’s trend review paper alone [20].

Unlike other materials, the performance of foams relies greatly upon their relative densities, which describe whether they are open-celled or close-celled. Consequently, future potential is difficult to gauge with one overarching materials property index. If it was assumed that it behaved as an open-cell foam exhibiting Euler buckling (with relative densities between 0.01 to 0.3), a more general criterion could be created based upon the goal of maximizing energy absorption at a minimum mass. In fact, Bird et al. modeled such a criterion (MPI_3_) during a case study on selecting the correct lightweight foam to make impact-absorbing helmets [72]. Here, E_S_ and ρ_S_ are the Young’s modulus and density of the solid material, respectively, which can be determined with knowledge of the foam’s relative density. Materials that optimize this index have high impact absorption at a minimum mass.

(3) $$\begin{array}{c} {\mathrm{MPI}}_{3}={E_{S}}^{0.729}/\rho_{s} \end{array}\begin{array}{c} \mathrm{Objective}:\;\mathrm{minimize}\;\mathrm{foam}\;\mathrm{mass}\;(\rho_{s})\\ \mathrm{Constraint}:\;\mathrm{high}\;\mathrm{enough}\;\mathrm{impact}\;\mathrm{energy}\;\mathrm{dissipation}\;({E_{s}}^{0.729}) \end{array}$$

If competitiveness is the plan, then future fungal foams must target an optimization of this index as compared to other cushioning foams (e.g., open-cell polyurethane, polyethylene, neoprene, etc.) [40,43,72]. Of course, this is only one of many factors for assessing viability, but it is a defined threshold of success. For now, however, the biggest obstacle in realizing the current potential of these foams is the unfortunate dearth of materials testing and literature studies.

### 3.4. Novel Prospects for Future Functional PMMs

There are several other functional PMMs under various stages of development. However, most are not industry ready, existing only within the laboratory. One particularly interesting niche is the study of mycelia as a biomaterial, building on the historical use of *Fomes fomentarius* and *Piptoporus betulinus* as bandaging materials [54,55]. Researchers have created therapeutic wound dressings out of the filamentous growths of select species with some encouraging results [73,74]. To mimic the extracellular matrix with the hyphal structure of mycelia, novel biomedical scaffolds have been developed with excellent physiochemical properties, all while being tunable and self-growing [75,76]. With growing interest in creating natural and customized biomaterials, PMMs could potentially fill the current gaps in tissue engineering. While mycelial biomaterials are an interesting proposition, in-depth testing of mechanical properties and biocompatibility is truly needed before these solutions can be implemented.

Another promising area of interest is the development of highly treated mycelium-derived films. These materials target a different category of materials beyond textiles and foams and offer a wide range of options. The majority of mycelial films are manufactured by harvesting biofilms from shaken liquid cultures which are then dried and treated with different chemicals such as glycerol [32,45]. Current explorations into film fabrication demonstrate their ability to replace a range of materials from similar natural materials to polymers and elastomers. As observed in Figure 2, films derived from the same *S. commune* species have drastically different tensile properties depending on the concentration of their glycerol treatment [45,51]. The lack of standardization in film fabrication demonstrates how different combinations of treatments and species offer their own unique advantages [33,45].

Nanopapers, another group at the periphery of the PMM family, are fabricated directly from the chitin–glucan and chitin–chitosan nanofibrils that make up mycelium [33,77]. In terms of stiffness or Young’s modulus, mycelial nanopapers treated with NaOH perform like industrial polymers and have higher densities (MPI_1_ = 5.72 Pa·m^3^/g) than most simple MBL and bovine leathers, while their elastic energy storage (MPI_2_ = 8.83 Pa·m^3^/g) is slightly lower [33]. It is abundantly clear that the performance of these nanopapers is highly variable depending on the species source of the materials, with worse performances by nanopapers derived from *Allomyces arbuscula* and *Trametes versicolor* [33]. These material properties charts offer only a glimpse at the bulk mechanical properties, but these nanopapers are purported to also have exceptional thermal and surface properties that can be tuned based on the treatment process [33]. It should be noted that while relative pure hyphal morphology was visible in the images of the *A. bisporus* nanopaper, nanofibrils could not clearly be identified at that scale [33]. All other species had much larger, micro-scale fibrils even after the remedial H_2_O_2_ or HCl treatments, which warrants some examination into the “nanopaper” title [33,76]. While these nanopapers are still in the early stages of conceptualization, chitin films derived from crustaceans with similar targets in biotechnological and coating applications have a firmer historical establishment and could offer a point of comparison [32,33,77,78]. The latest of these chitin films boast similar claims of tunability with the ability to demonstrate high tensile strength (up to 226 MPa) or high elongation (up to 43%) depending on the choice of chemical cross-linking [78,79,80]. Future development of mycelial nanopapers must emphasize the advantages of their hyphal-structured nanofibrils in order to grant clearer product differentiation beyond just fungi-derived chitin films.

## 4. Leveraging Unique Species for PMMs

### 4.1. Functional Species of Fungi

With around 150,000 identified species of fungi, there is a diverse spread of hyphal morphologies and growth characteristics from which to create functional fungal materials [2,20]. Species producing edible or medicinal mushrooms are routinely investigated due to the wealth of pre-existing knowledge about optimal growth conditions and cultivating these species. However, there is untapped potential in other species that are not ordinarily of note due to the fruiting bodies being too tough, like the Amadou or horse hoof fungus *(Fomes fomentarius*), or simply inedible, like birch polypore *(Piptoporus betulinus*) [54,81]. The steady rise of mycelial materials has encouraged the investigation of these and other unknown species of fungi across several phyla to find suitable candidates for new materials development [82] (Table 3).

One of the prominent trends in Table 3 was the chosen production method and substrates among different PMMs. Out of the total 94 observed PMMs, there were 15 produced through SSF, 50 produced through LSF (6 SmF and 44 LSSF), and 15 produced through specialized processes. There were also 3 PMMs developed through patented methods by MycoWorks, Grado Zero Innovation, and Ecovative LLC [61,62,85]. While the method of fermentation was divided between LSF and SSF, there was no ubiquitous nutritional media for either case, making it difficult to compare between species, including their advantages and potential problems. However, it was evident that LSF was more popular due to the relative homogeneity of liquid media and the ease of harvesting the PMM. PMMs derived from fruiting bodies were made either directly, to make Amadou and similar products, or indirectly, to extract chitin by grinding. Another process included the mycelium isolation of a non-dikaryotic species called *Rhizopus stolonifera*. The sporangia-producing mucoromycete was studied for the creation of a thick “mycelium mattress” that was deproteinized and dried for wound healing purposes [2,111].

In total, over sixty distinct species of fungi have been investigated for pure mycelial materials application, each with their own sets of advantages and disadvantages. Along with well-known medicinal species like *Ganoderma lucidum* and *Trametes versicolor*, other species such as *Phellinus ellipsoideus* and *F. fraxinea* have shown excellent potential for creating mycelial materials with quick growth and dense hyphal structures [19,85,113]. The group of 64 species spanned 4 different phyla, including 55 basidiomycetes, 2 ascomycetes, 6 mucoromycetes, and 1 blastocladiomycete, each characterized by their unique hyphae. Basidiomycota and Ascomycota, both classified as ‘higher fungi’ under the Dikarya subkingdom, are the two most species-rich phyla and contain mostly filamentous species [4]. Many dikaryotic species allow for the formation of large fruiting bodies with varied and complex hyphal structures known as sporocarps [2,4]. On the other hand, Mucoromycota and Blastocladiomycota do not produce large fruiting bodies [2]. Nevertheless, these ‘lower fungi’ can also provide rapid mycelial growth to create dense mats for medical applications or produce large quantities of chitin for nanopaper fabrication [33,73,111].

**Figure 5:**
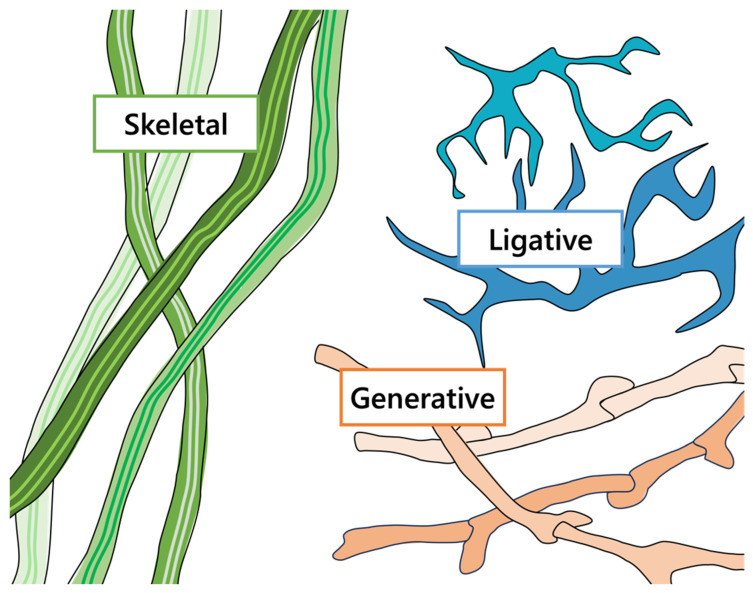
The common types of hyphae used to classify the hyphal systems of basidiocarps: generative hyphae with clamp connections (orange); unbranched, continuous skeletal hyphae (green); and highly branched, binding ligative hyphae (blue).

Within the vast phyla of Basidomycota in particular, there comes a need to classify them concisely to separate the softer mushrooms (e.g., *Agaricus bisporus*, *Pleurotus ostreatus*, etc.) from the woody brackets (e.g., *Fomes fomentarius*, *Ganoderma applanatum*, etc.). The hyphal structures of these species greatly influence the morphological, mechanical, and physiochemical characteristics of the resultant fungal material [81,113,114]. In total, the mushroom or basidiocarps consist of three main types of hyphae: generative, skeletal, and ligative, as shown in Figure 5 [4,115]. Generative hyphae are thin walled, branching, and separated with septa [115]. Clamp connections, unique to some species in Basidiomycota, are hook-like protrusions near the septa of generative hyphae that develop during the process of sexual mating [4,115]. During mushroom growth, generative hyphae can transition into either of the thick-walled skeletal or ligative hyphae over time [115]. Skeletal hyphae are elongated, unsegmented strands which tend to overlap one another [4,115]. With their characteristic unidirectional growth, skeletal hyphae serve as the matrix in many species of fungi [115]. Lastly, ligative or binding hyphae are unsegmented and branching, characterized by a curling and gnarled structure [4,115]. As the name implies, the binding hyphae tightly holds together the mushroom’s shape, which offers considerable mechanical strength and stiffness [114,115].

Depending on species, these types of hyphae are found at varying levels [113,114]. Mushrooms containing only generative hyphae are monomitic, while species with all three are trimitic. Out of the 55 basidiomycetes listed in Table 3, 15 were monomitic, 14 were dimitic, and 17 were trimitic, while the remaining were difficult to classify. Dimitic systems always contain generative hyphae, commonly combined with skeletal and rarely with ligative hyphae. Bracket fungi, which are typically trimitic and therefore stiffer than other species, have been identified as particularly promising. Beside morphology, hyphal systems impact the mechanical and fluid absorption properties of the mushroom [113,116]. Previous investigations have demonstrated that hyphal structure of mushrooms can be leveraged when choosing between divergent species in the design of PMMs [81,114,117]. Moreover, these studies have also shown that sturdy mushrooms, in their virgin state, can themselves serve as cheap, ultralightweight materials for design and construction.

### 4.2. Tailoring Bespoke Mycelium with Strain Optimization

Along with capitalizing on the intricacies of different hyphal systems, species phenotyping offers more tools to optimize mycelial material fabrication. Ubiquitous among all filamentous fungi, the porous morphology of mycelium is characterized by random hyphal networks rich in chitin, β-glucans, and other glycoproteins [3,118]. The polymer composition and skeletal structure of inner cell walls are actually quite conserved in the majority of species, and it is instead the variable organization of outer cell walls that dictates morphology and behavior between species [3]. Chitin contributes stiffness, while the β-glucans, with their spring-like shape, offer tensile strength to the cells [3,119]. The hydrophobins, another group of cell wall proteins, are found on the outer surface of the hyphal cell walls and can repel water from the mycelial structures [120]. Studies on *S. commune* have shown that its *SC3* hydrophobin gene, among others, actually plays an active role in reducing surface tension and encourages the growth of aerial hyphae [121,122]. In some strains where *SC3* expression is disrupted, both the formation of aerial hyphae and the mycelium’s attachment to the hydrophobic surface are reduced [121,123]. Identifying the cell wall-related genes had been shown to be a great tool in understanding the structure and signaling pathways of pathogen fungi such as *Aspergillus fumigatus* but could also offer the opportunity to create tailored strains to fit PMM mechanical needs [104,124].

There has been recent interest in leveraging this gene deletion for the cultivation of denser mycelial mats by examining the effects of gene disruption alongside altered environmental conditions such as CO_2_ and light levels [104]. In fact, deletion of the *SC3* gene in *S. commune* (*∆sc3* strain) causes a drastic increase in the density, Young’s modulus, and tensile strength of the mycelial mat as compared to a wild-type strain. These new properties of the *∆sc3* mycelia are similar to those of polymers, while those of the wild type strain are more in line with natural materials such as cork or bamboo [104]. Species such as the button mushroom *Agaricus bisporus* and the oyster mushroom *Pleurotus ostreatus* have comparative hydrophobin genes, while others like *Ustilago maydis* have amphipathic peptides named ‘repellents’ that, when deleted, reduce aerial hyphae formation [125,126,127]. Borrowing the techniques used to genetically engineer *S. commune* to produce mycelial materials could drive innovation towards perfecting mechanical properties and identifying specialized species for each PMM application. Ecovative LLC has already experimented with genetic engineering by introducing *CDA1*, the chitin deacetylase-encoding gene in *Saccharomyces cerevisiae*, to production strains as a method of increasing the compression strength of CMMs [128]. These prospective technologies aim to combine genetic engineering and materials science at the cutting-edge of mycelial material development.

The potential advantages of strain optimization deserve greater attention in order to truly redefine the biological upper bounds of mycelial thickness and stiffness and perhaps target loftier ambitions by emulating materials such as elastomers and rubbers. However, it is important to note that while genetic engineering can drastically improve mechanical properties, it could also lead to a cascade of unintended effects on antibiotic resistance, virulence, and influence on natural populations [129]. As such, the approaches to this strategy must be carefully controlled, especially in the creation of edible PMMs and mycoproteins. With a wide span of knowledge gaps in genetic engineering and industrial production, the physical upper bounds of PMMs are much more unknown and will require a coordinated effort to uncover.

## 5. Conclusions

More than ever, the successful portfolio of pure mycelial materials demonstrates how adaptable the chitin–glucan structure of fungal mycelia is for making lightweight yet durable leather alternatives to tunable high-performance foams. Future efforts are presumed to focus on further improving the mechanical properties of these flexible materials. With new materials and fundamental biological discoveries about fungi on the horizon, there is great potential to optimize SSF and LSF to meet the challenges. Following scaling up for industrial applications, the chance of contamination could increase, and further optimizations of environmental conditions are needed. How these concerns are answered in the coming years will be key in determining the future feasibility of these greener materials supplanting the more established competitors. Finally, with current efforts to diversify the material selection pool of fungal species and in combination with our expanding knowledge about pathogenic and toxigenic fungi [130], there will be better understanding of what makes a species a successful progenitor of hyphal structure and mycelial growth for humans. Ultimately, could genotyping of these species eventually allow for growth and mechanical properties to be handpicked for the creation of bespoke mycelial materials through genetic modification? There are many outstanding questions and challenges about the future of this field, but only with a successful dissemination of current knowledge can the process to unravel them begin.

## Figures and Tables

**Table 1:** Common properties of materials, their definitions, and their units.

Materials Property	Definition	Unit
Density (ρ)	A material’s mass per unit of volume.	kg/m^3^
Percent elongation (%EL)	A material’s deformation when it fractures due to a tensile load.	%
Ultimate tensile strength (σ_UTS_)	The maximum amount of strength a material can withstand under tension.	Megapascals (MPa)
Young’s modulus (E)	The modulus of elasticity or the material’s ability to stretch and deform.	Gigapascals (GPa)

**Table 2:** Physical properties of mycelium-based leathers versus conventional leathers.

Textile	ρ (kg/m^3^)	%EL	σ_UTS_ (MPa)	E (GPa)	MPI_1_ (Pa·m^3^/g)	MPI_2_ (Pa·m^3^/g)	References
*Fomitella fraxinea* MBL (oak & bran substrate)	1580	4.30–4.98	1.18–1.62	0.00117–0.00157	0.0875	893	[19]
*Fomitella fraxinea* MBL polyethylene glycol treated with 120 °C heat press (oak & bran substrate)	1460	13.87–17.91	6.28–8.14	0.00669–0.00736	4.9	4990	[19]
*Rhizopus delemar* MBL (bread substrate)	884–922	1.7–2.3	19.04–20.74	1.38–1.50	2.2	304	[7]
*Rhizopus delemar* MBL, tannin/glycerol treated (bread substrate)	695–739	14.5–18.6	2.51–2.93	0.199–0.201	0.378	5.14	[7]
MuSkin^TM^ MBL (Grado Zero Innovation, Firenze, Italy) *	1000	17.3–38.6	0.3–0.4	0.0018–0.0028	0.0346	53.5	[52,62]
Reishi^TM^ Brown Natural MBL (MycoWorks, Emeryville, CA, USA) *	480–540	16–36	5.6–7.4	-	1.27	-	[46,63]
Reishi^TM^ Brown Natural High Strength MBL (MycoWorks) *	8400	55–80	8.8–12.5	-	0.125	-	[47,63]
Reishi^TM^ Black Emboss MBL (MycoWorks) *	740–880	51–52	9.2–10.2	-	1.2	-	[48,63]
Mylea^TM^ MBL (Mycotech; Aurora, CO, USA) *	1330–4440	22–35	8–11	-	0.386	-	[49,50]
Artificial leather (Polyurethane composites)	340–470	-	9.4–24.5	0.012–0.036	12	87,700	[52,64,65]
Bovine leather	810–1050	18–75	20–50	0.10–0.50	3.43	4560	[51]

MPI_1_ highlights materials strong enough to resist tensile rupture (σ_UTS_) that also meet the objective of minimum mass (ρ), which is measured in (Pa·m^3^/g). MPI_2_ highlights materials with sufficiently high elastic energy storage (σ_UTS_^2^/E) that also meet the objective of minimum mass (ρ), which is measured in (Pa·m^3^/g). * indicates that the MBL is a commercial product.

**Table 3:** Summary of fungal species investigated as pure mycelial materials. This table lists unique species that have been investigated as pure mycelial materials over the last 30 years. Species are written as they are reported in the original works. Additionally, species used solely for composite mycelial materials are omitted here.

Application	Species	Cultivation	Substrate	References
Amadou	*Fomes fomentarius* ^IIIb^	natural growth	beech, birch, oak, poplar, willow, and maple trees	[54,55,83]
	*Piptoporus betulinus* ^IIb^	natural growth	birch trees	[54,84]
Flexible foam	*Ganoderma* sp. ^b^	SSF	corn stover, grain spawn, maltodextrin, calcium sulfate, and minerals	[6]
	*Trametes versicolor* ^IIIb^	SSF	proprietary fabrication by Ecovative LLC.	[83,85]
Leather	*Abortiporus biennis* ^Ib^	LSF (LSSF)	homogenized millet slurry	[18,86]
	*Bjerkandera adusta* ^Ib^	LSF (LSSF)	homogenized millet slurry	[18,87]
	*Bjerkandera adusta* ^Ib^	SSF	oak sawdust and rice bran	[19,87]
	*Coriolopsis gallica* ^IIIb^	LSF (LSSF)	homogenized millet slurry	[18,83]
	*Coriolopsis trogii* ^IIIb^	LSF (LSSF)	homogenized millet slurry	[18,83]
	*Daedaleopsis confragosa* ^IIIb^	LSF (LSSF)	homogenized millet slurry	[18,83]
	*Daedaleopsis tricolor* ^IIIb^	LSF (LSSF)	homogenized millet slurry	[18,83]
	*Elfvingia applanate* ^IIb^	SSF	oak sawdust and rice bran	[19,88]
	*Fomes fomentarius* ^IIIb^	LSF (LSSF)	homogenized millet slurry	[18,83]
	*Fomitella fraxinea* ^b^	SSF	oak sawdust and rice bran	[19]
	*Fomitiporia mediterranea* ^IIb^	LSF (LSSF)	homogenized millet slurry	[18,89]
	*Fomitopsis iberica* ^IIIb^	LSF (LSSF)	homogenized millet slurry	[18,90]
	*Fomitopsis pinicola* ^IIIb^	LSF (LSSF)	homogenized millet slurry	[18,83]
	*Fomitopsis pinicola* ^IIIb^	SSF	oak sawdust and rice bran	[19,83]
	*Fomitopsis rosea* ^II-IIIb^	SSF	oak sawdust and rice bran	[19,91]
	*Ganoderma applanatum* ^IIIb^	SSF	oak sawdust and rice bran	[19,88,92]
	*Ganoderma carnosum* ^b^	LSF (LSSF)	homogenized millet slurry	[18]
	*Ganoderma lucidum* ^IIIb^	SSF	proprietary fabrication by MycoWorks	[61,88,92]
	*Ganoderma lucidum* ^IIIb^	LSF (LSSF)	homogenized millet slurry	[18,88,92]
	*Ganoderma lucidum* ^IIIb^	SSF	oak sawdust and rice bran	[19,88,92]
	*Irpex lacteus* ^IIb^	LSF (LSSF)	homogenized millet slurry	[18,93]
	*Irpiciporus pachyodon* ^Ib^	LSF (LSSF)	homogenized millet slurry	[18,94]
	*Lenzites betulinus* ^IIIb^	LSF (LSSF)	homogenized millet slurry	[18,83]
	*Microporus affinis* ^IIIb^	SSF	oak sawdust and rice bran	[19,87]
	*Neofavolus alveolaris* ^IIb^	LSF (LSSF)	homogenized millet slurry	[18,95]
	*Phellinus ellipsoideus* ^b^	-	proprietary fabrication by Grado Zero Innovation	[62]
	*Pleurotus ostreatus* ^Ib^	LSF (LSSF)	Czapek medium	[17,96]
	*Postia balsamea* ^Ib^	SSF	oak sawdust and rice bran	[19,97]
	*Rhizopus delemar* ^d^	LSF (LSSF)	bread waste	[7]
	*Stereum hirsutum* ^IIb^	LSF (LSSF)	homogenized millet slurry	[18,98]
	*Terana caerulea* ^Ib^	LSF (LSSF)	homogenized millet slurry	[18,99]
	*Trametes hirsuta* ^IIIb^	LSF (LSSF)	homogenized millet slurry	[18,83]
	*Trametes hirsuta* ^IIIb^	SSF	oak sawdust and rice bran	[19,83]
	*Trametes suaveolens* ^IIIb^	LSF (LSSF)	homogenized millet slurry	[18,83]
	*Trametes suaveolens* ^IIIb^	SSF	oak sawdust and rice bran	[19,83]
	*Trametes versicolor* ^IIIb^	SSF	oak sawdust and rice bran	[19,83]
	*Wolfiporia extensa* ^b^	SSF	oak sawdust and rice bran	[19]
Mycelial film	*Aurantiporus* sp. ^b^	LSF (LSSF)	potato dextrose broth	[32]
	*Coriolus brevis* ^b^	LSF (LSSF)	potato dextrose broth with glycerol and skim milk	[39]
	*Coriolus hirsutus* ^b^	LSF (LSSF)	potato dextrose broth with glycerol and skim milk	[39]
	*Coriolus versicolor* ^b^	LSF (LSSF)	potato dextrose broth with glycerol and skim milk	[39]
	*Fomitella fraxinea* ^b^	LSF (LSSF)	potato dextrose broth with glycerol and skim milk	[39]
	*Ganoderma curtisii* ^IIIb^	LSF (LSSF)	potato dextrose broth	[32,92]
	*Ganoderma lucidum* ^IIIb^	LSF (LSSF)	potato dextrose broth with d-glucose and lignin	[88,92,100]
	*Ganoderma lucidum* ^IIIb^	LSF (LSSF)	potato dextrose broth with glycerol and skim milk	[39,88,92]
	*Ganoderma mexicanum* ^IIb^	LSF (LSSF)	potato dextrose broth	[32,101]
	*Lentinus crinitus* ^IIb^	LSF (LSSF)	potato dextrose broth	[32,102]
	*Panus conchatus* ^I-IIb^	LSF (LSSF)	potato dextrose broth	[32,103]
	*Pleurotus ostreatus* ^Ib^	LSF (LSSF)	potato dextrose broth	[32,96]
	*Polyporus arcularius* ^IIb^	LSF (LSSF)	potato dextrose broth with glycerol and skim milk	[39,83]
	*Polyporus squamosus* ^IIb^	LSF (LSSF)	potato dextrose broth with glycerol and skim milk	[39,83]
	*Pycnoporus coccineus* ^IIIb^	LSF (LSSF)	potato dextrose broth with glycerol and skim milk	[39,83]
	*Schizophyllum commune* ^b^	LSF (SmF)	minimal media with glucose and asparagine	[45,104]
	*Trametes fuciformis* ^b^	LSF (LSSF)	potato dextrose broth with glycerol and skim milk	[39]
	*Trametes gibbosa* ^b^	LSF (LSSF)	potato dextrose broth with glycerol and skim milk	[39]
	*Trametes orientalis* ^b^	LSF (LSSF)	potato dextrose broth with glycerol and skim milk	[39]
	*Inonotus obliquus* ^Ib^	LSF (SmF)	potato dextrose broth	[34,105]
Nanofilms	*Agaricus bisporus* ^Ib^	mushroom isolation	-	[77,106]
	*Grifola frondosa* ^IIb^	mushroom isolation	-	[77,107]
	*Hypsizygus marmoreus* ^b^	mushroom isolation	-	[77]
	*Lentinula edodes* ^Ib^	mushroom isolation	-	[77,108]
	*Pleurotus eryngii* ^Ib^	mushroom isolation	-	[77,96]
	*Tricholoma terreum* ^b^	mushroom isolation	-	[109]
Nanopapers	*Agaricus bisporus* ^Ib^	LSF (SmF)	diluted blackstrap molasses	[33,106]
	*Allomyces arbuscula* ^c^	LSF (SmF)	diluted blackstrap molasses	[33]
	*Mucor genevensis* ^d^	LSF (SmF)	diluted blackstrap molasses	[33]
	*Trametes versicolor* ^IIIb^	LSF (SmF)	diluted blackstrap molasses	[33,83]
Scaffold	*Aspergillus* sp. ^a^	LSF (LSSF)	potato dextrose broth	[76]
	*Pleurotus ostreatus* ^Ib^	LSF (LSSF)	potato dextrose broth	[75,96]
	*Trametes versicolor* ^IIIb^	LSF (LSSF)	potato dextrose broth	[75,83]
Tinder	*Fomes fomentarius* ^IIIb^	natural growth	beech, birch, oak, poplar, willow, and maple trees	[54,83]
Wound dressing	*Agaricus bisporus* ^Ib^	stipe isolation	-	[73,106]
	*Fomes fomentarius* ^IIIb^	natural growth	beech, birch, oak, poplar, willow, and maple trees	[54,55,83]
	*Fusarium graminearum* ^a^	mycelium isolation	-	[73]
	*Phanerochaete chrysosporium* ^IIb^	LSF (LSSF)	malt, dextrose, and peptone broth	[74,110]
	*Phycomyces blakesleeanus* ^d^	spore isolation	-	[73]
	*Rhizomucor miehei* ^d^	mycelium isolation	-	[73]
	*Rhizopus oryzae* ^d^	mycelium isolation	-	[73]
	*Rhizopus stolonifer* ^d^	mycelium isolation	potato dextrose agar	[111]

The hyphal system of each species is indicated by I, II, and III for monomitic, dimitic, and trimitic, respectively. The phylum of each species is indicated by a, b, c, or d for *Ascomycota*, *Basidiomycota*, *Blastocladiomycota*, and *Mucoromycota* (previously *Zygomycota*); each species was classified by phyla based on the Global Biodiversity Information Facility database [2,112].

## Data Availability

Data are contained within the article.

## Abbreviations

CMMs	composite mycelial materials
GPa	gigapascals
LSF	liquid-state fermentation
LSSF	liquid-state surface fermentation
MBLs	mycelium-based leathers
MPa	megapascals
MPI	material property index
PEG	polyethylene glycol
PMMs	pure mycelial materials
SEM	scanning electron microscope
SmF	submerged fermentation
SSF	solid-state fermentation
